# One‐carbon metabolism modulates *miR‐29a*–DNA methylation crosstalk in Alzheimer's disease

**DOI:** 10.1002/alz.70703

**Published:** 2025-09-23

**Authors:** Tiziana Raia, Rosaria A. Cavallaro, Luiza Diniz Ferreira Borges, Stefano Cinti, Mariano Bizzarri, Isidre Ferrer, Marco Lucarelli, Andrea Fuso

**Affiliations:** ^1^ Department of Experimental Medicine Sapienza University of Rome Rome Italy; ^2^ Department of Surgery Sapienza University of Rome Rome Italy; ^3^ Department of Pharmacy University of Naples “Federico II” Naples Italy; ^4^ Department of Pathology and Experimental Therapeutics Emeritus Professor University of Barcelona Hospitalet de Llobregat Barcelona Spain; ^5^ Pasteur Institute Cenci Bolognetti Foundation Sapienza University of Rome Rome Italy; ^6^ CRiN Center for Research in Neurobiology Sapienza University of Rome Rome Italy

**Keywords:** Alzheimer's disease, DNA methylation, epigenetics, microRNAs, *mir‐29a*, non‐CPG methylation, one‐carbon metabolism

## Abstract

**INTRODUCTION:**

Alzheimer's disease (AD)’s multifactorial nature stresses the role of epigenetics in affecting different pathological pathways. We demonstrated that one‐carbon metabolism epigenetically impacts AD‐like phenotype. Here, we investigated the crosstalk between methylation and microRNAs in AD.

**METHODS:**

We altered one‐carbon metabolism to induce hypo‐ and hyper‐methylation, in SK‐N‐BE neuroblastoma cells and TgCRND8 mice. miRNAs were profiled through a polymerase chain reaction array, then we focused on *miR‐29a* expression and methylation of its genomic locus. Finally, we assessed *miR‐29a* expression and methylation in the brain of AD subjects.

**RESULTS:**

*MiR‐29a* was repressed in hypomethylating and expressed in hypermethylating conditions. The expression of *miR‐29a* and of its target, *BACE1*, was inversely correlated.

**DISCUSSION:**

We demonstrated for the first time that *miR‐29a* is modulated by one‐carbon metabolism through DNA methylation, disclosing the molecular mechanisms regulating *BACE1* expression in AD. These data confirm *miR‐29a*’s protective role in AD and support *miR‐29a* as a potential biomarker for AD.

## 1 BACKGROUND

The sporadic, late‐onset, form of Alzheimer's disease (LOAD) is the prevalent form of dementia, and it is known to have multifactorial basis.^1^, ^2^, ^3^ LOAD is characterized by different pathological hallmarks, including amyloid beta (Aβ) processing and deposition,^4^ tau hyperphosphorylation and accumulation,^5^ oxidative stress,^6^, ^7^ and neuroinflammation.^7^, ^8^ These processes involve multiple molecular pathways, contributing to AD pathophysiology.^9^, ^10^ Previous studies suggest that the multifactorial nature of LOAD can hide epigenetic regulation of the above‐mentioned molecular pathways.^11^, ^12^, ^13^, ^14^ Building on clinical data evidencing impaired methylation potential (MP) in the elderly and those with AD,^15^, ^16^ we studied the role of DNA methylation—the most studied epigenetic factor—in the amyloidogenic process.^17^, ^18^ MP represents the ratio between S‐adenosylmethionine (SAM, the methyl donor for many substrates, including DNA) and S‐adenosylhomocysteine (SAH), the byproduct of the transmethylation reactions, that inhibits the methyltransferases.^19^ We developed an experimental approach consisting of SAM supplementation to enhance MP, and B vitamin deficiency (B12, B6, and folate), to impair it.^20^ B vitamins are cofactors in the reactions that allow the transformation of homocysteine (HCY) produced by SAH hydrolysis:^21^ if HCY is not removed, SAH accumulates, impairing MP. These reactions are part of the so‐called “one‐carbon metabolism.”^21^ We demonstrated, in neuronal cells and in AD transgenic mice, that B vitamin deficiency upregulates β‐ and γ‐secretases through overexpression of their genes: respectively, *BACE1* and *PSEN1*.^20^ SAM supplementation restored control‐like expression levels of these genes.^20^ Notably, *PSEN1* upregulation was directly correlated to the hypomethylation of its promoter,^22^ while *BACE1* promoter methylation did not show significant changes in response to one‐carbon metabolism modulation,^23^ suggesting the involvement of a “mediator” of the epigenetic regulation.

- *miR‐29a* targets *BACE1* mRNA reducing β‐secretase expression and amyloidogenesis in Alzheimer's disease.
- One‐carbon metabolism modulates *PSEN1*, via DNA methylation, and *BACE1*, via *miR‐29a*.
- Non‐CpG hypermethylation of the *miR‐29a* sequence is associated to *miR‐29a* upregulation.
- miR‐29a regulation indicates a crosstalk between methylation and microRNAs.
- One‐carbon metabolism exerts a pleiotropic effect on amyloidogenesis.

We therefore considered the role of microRNAs (miRNAs) in AD. They are considered epigenetic factors that modulate gene expression primarily by binding to a complementary mRNA sequence, thereby inhibiting translation or inducing degradation. Characterized by a length of ≈ 22 to 25 nucleotides, miRNAs retain at least one conserved binding site hosted by > 60% of human protein‐coding genes.^24^ miRNAs can target specific mRNA sequences, fine‐tuning the expression of genes. The intricate crosstalk among DNA methylation, histone modifications, and miRNAs is crucial to clarify how alterations in these mechanisms contribute to various diseases, including cancers and neurodegenerative disorders.^25^, ^26^ Their coordinated actions orchestrate the dynamic regulation of gene expression essential for proper cellular function and development. In the specific AD context, characterized by alterations in multiple molecular pathways, it appears essential to adopt a multi‐parametric approach.^27^ In this perspective, one‐carbon metabolism may play a pivotal role in regulating amyloidogenesis through complementary molecular mechanisms.

**RESEARCH IN CONTEXT**

1. **Systematic review**: One‐carbon metabolism (OCM) epigenetically modulates Alzheimer's disease (AD)‐like symptoms through *PSEN1* and *BACE1* regulation. *PSEN1* expression is directly modulated by promoter methylation. *BACE1* is dependent on methylation potential but independent on promoter methylation. Looking for mediators connecting methylation and *BACE1*, we investigated how OCM modulates AD‐associated miRNAs. B vitamin deficiency and S‐adenosylmethionine supplementation influence OCM inducing, respectively, hypo‐ and hypermethylation.
2. **Interpretation**: The cellular methylation potential modulates *BACE1* through *miR‐29a*. This also confirms the *miR‐29a* protective role in AD. These data place OCM and methylation reactions as pivotal epigenetic regulators of amyloidogenesis, acting in parallel on *PSEN1* and *BACE1*. DNA methylation seems therefore a relevant modifier in AD onset and progression, with a particular emphasis on non‐CpG methylation.
3. **Future directions**: OCM and methylation play a central role in AD, because they also are involved in amyloid scavenging, trafficking, tau phosphorylation, neuroinflammation, and oxidation. More ample research is therefore warranted. microRNAs and DNA methylation represent potential AD biomarkers.

Many different miRNAs have been recognized as relevant to AD^28^, ^29^, ^30^ with detrimental^31^, ^32^, ^33^ or protective roles.^34^, ^35^, ^36^

One of the most extensively studied is the *miR‐29a*, widely considered a “protective” factor in neurodegenerative processes. The loss of *miR‐29a*/*b‐1* has been associated with AD, providing a potential causal relationship between miRNA expression and Aβ formation primarily due to its role in targeting *BACE1* mRNA.^37^, ^38^, ^39^, ^40^

The gene encoding the precursor of *miR‐29a* and *miR‐29b‐1* is located on chr. 7q32.3 in humans and on chr. 6 in mice. *miR‐29a* seems to be co‐transcribed with *miR‐29b‐1* as a polycistronic primary transcript.^41^, ^42^ The *miR‐29a/b‐1* cluster is hosted by the long‐non‐coding RNA (lncRNA), *LOC646329* (also known as *MIR29HG*).^43^

The DNA methylation–miRNAs crosstalk is particularly relevant for the *miR‐29* family, as it is implicated in epigenetic regulation, by directly targeting DNMT3A and DNMT3B.^44^ *miR‐29* family members repress two other key components of the DNA methylation machinery, namely ten‐eleven traslocation 1 (TET1) and thymine DNA glycosylase (TDG). Thus, a loss of *miR‐29* functionality could lead to aberrant methylation level.^45^

We took advantage of a miRNA polymerase chain reaction (PCR) array to assess whether hyper‐ or hypomethylating conditions could regulate the expression of specific miRNAs. Among the significantly regulated miRNAs, we focused on *miR‐29a* due to its involvement both in AD and in DNA methylation processes. We then characterized its expression in human neuroblastoma cells and in the brains of AD mice, in relation to DNA methylation.

## 2 METHODS

### 2.1 Cell cultures

SK‐N‐BE cells (a human neuroblastoma cell line) were maintained in F14 medium (prepared in‐house) supplemented with 10% fetal bovine serum (FBS), and antibiotics (penicillin 100 IU/mL, streptomycin 100 µg/mL) at 37°C, 5% CO_2_ and 80% humidity. B vitamin deficient (B‐def) F14 medium was prepared as the complete medium but without B6, B12, and folate. All the reagents were from the Merck Group.

According to the experimental design, cells were seeded in complete F14 medium with 10% FBS; after 24 hours of growth (T0), the cells were shifted to F14 1% FBS complete (Ctrl) or B‐def, with or without SAM 100 µM (Ctrl+SAM and B‐def+SAM). SAM was obtained from Gnosis by Lesaffre. Cells were stopped after 48 hours for DNA methylation assay and after 96 hours for gene expression analysis.

### 2.2 In vivo experiments

TgCRND8 transgenic mice (kindly provided by D. Westaway, University of Toronto) overexpress a double mutant form of human amyloid precursor protein 695 (APP695, carrying the “Swedish” KM670/671NL and the “Indiana” V717F mutations). The 5‐fold higher transgene expression respect to the endogenous AβPP causes the age‐dependent accumulation of “human” Aβ40 and Aβ42, with the toxic Aβ42 being the more prevalent.^46^ Heterozygous transgenic mice (TgCRND8^+/−^) and wild‐type (129 Sv, Wt) littermates, used as controls, were obtained from the same matings. Genotype was assessed on tail fragments of pups as previously described.^20^

Animals were housed in an air‐conditioned room (temperature 21 ± 1°C, relative humidity 60 ± 10%) with a 12:12 hour light:dark cycle (lights on from 8:00 a.m. to 8:00 p.m.) and food and water continuously available. SAM was premixed to the complete or B‐deficient diet pellets (Mucedola s.r.l.) at a concentration of 0.1 g/kg to provide a SAM dosage of 400 µg/day as previously described.^20^

An equal number of female and male mice were randomly assigned, at weaning (postnatal day [PND] 21) to control, Ctrl+SAM, B‐def, or B‐def+SAM diet groups (*N* = 20 for each group). No sex‐associated differences have been observed here or in previous experiments. At 3 months of age (PND 90), brain tissues were collected as previously described^20^ and mechanically homogenized (TissueLyser II, Qiagen) in the appropriate buffers for DNA or RNA purification. All procedures were carried out according to the European Communities Council Directives (86/609/EEC and 2010/63/EU) and Italian national legislation on animal experimentation (D.Lgs 116/92) and formally approved by the Italian Ministry of Health.

### 2.3 Human subjects

Human brain tissues were obtained and used in compliance with the Declaration of Helsinki, and the relevant Spanish laws provided by the medical ethics committees. The study was approved by the local ethics committee.

Samples included brain tissues (frontal cortex, *N* = 15 per group) from middle‐aged healthy subjects (MA, mean age 69.3) and individuals with LOAD at Braak stages I to II/A (mean age 71.1) and V to VI/C (mean age 67.8). These samples were sourced from the Institute of Neuropathology Brain Bank (HUB‐ICO‐IDIBELL Biobank, Barcelona, Spain) and the Clinic Hospital‐IDIBAPS Biobank (Barcelona, Spain). Details of the tissue collection protocol and characterization of control and LOAD cases are provided elsewhere.^22^ The brain tissues were homogenized as above described.

### 2.4 miRNAs profiling

RNA from SK‐N‐BE cells was isolated using the RNeasy Plus Mini Kit (Qiagen) as previously described.^47^ The nucleic acid concentration was measured using a Nanodrop spectrophotometer. miRNA profiling was performed with the Human Cancer Pathway Finder miScript miRNA PCR array (Qiagen), containing specific forward primers. Data were analyzed through the Kyoto Encyclopedia of Genes and Genomes (KEGG) and Gene Ontology (GO) pathway enrichment tools using the Diana‐miRPath v2.0 software (Qiagen). To generate miRNA‐enriched cDNA, 125 ng of RNA was reverse‐transcribed using miScript RTII kit (Qiagen). One µg of total cDNA was used for quantitative PCR (qPCR) with the CFX Connect Real‐Time PCR Detection System (Bio‐Rad Laboratories) and miScript SYBR Green PCR kit (Qiagen).

### 2.5 Reverse transcription qPCR

RNA was extracted from cells as described above and from homogenized brain tissue using the RNeasy Lipid Tissue mini kit (Qiagen).^20^, ^23^

For the assessment of specific miRNA and mRNA expression by reverse transcription (RT)‐qPCR, cDNA was prepared as above described for the miRNAs, and by using the iScript cDNA Synthesis Kit (Bio‐Rad Laboratories) for mRNAs. qPCR was performed using the iTaq Universal SYBR Green (Bio‐Rad Laboratories). *BACE1*, *TET1*, and *TNFR1* mRNA levels were normalized to β‐actin (BACT) and expressed as the fold‐increase over control samples (i.e., Ctrl medium/diet or MA samples). GAPDH and 18S were also used as housekeeping genes, yielding results consistent with BACT (data not shown). All analyses were performed in triplicate. The primers used in qPCR are listed in Table 1.

**TABLE 1:** Sequences of the oligonucleotides used as polymerase chain reaction (PCR) primers for RNA expression in real‐time PCR (up) and for DNA amplification after bisulfite modification (bottom).

GENE	FORWARD PRIMER (5’ ‐ 3’)	REVERSE PRIMER (5’ ‐ 3’)
Real‐time PCR primers		
*BACE1*	AACGAATTGGCTTTGCTGTC	AGCCACAGTCTTCCA GTCC
*TET1*	GCAGCGTACAGGCCACCACT	AGCCGGTCGGCCATTGGAAG
*TNFR1*	ACAAGCCACAGAGCCTAGACACTG	ACGAATTCCTTCCAGCGCAACG
*β‐ACTIN*	CAACCGCGAGAAGATGACC	AGAGGCGTACAGGGATAGCA
*GAPDH*	TGGGATTTCCATTGACAAGC	CCCTTCATTGACCTCAACTACATG
*miR‐29a*	TAGCACCATCTGAAATCGGTTA	mRQ (Universal TAKARA)
*RNU6*	Primer Fw kit Takara	mRQ (Universal TAKARA)
*SNORD48*	CGTGATGACATTCTCCGGAATC	mRQ (Universal TAKARA)
Bisulfite assay PCR primers		
HSMIR29Bis	AATAAAYYTATAGYAYTTAATAGATA	ATRRAATTTCTATAAARTATAACCAT
MMMIR29Bis	TAATGAAAGTYAAGTTTAAGATAGGA	TACTTRTTCATRTAATAARCCTTCT

### 2.6 miR‐29a silencing and overexpression

SK‐N‐BE human neuroblastoma cells were seeded in six‐well plates 1 day before transfection, to achieve ≈ 60% confluence. These cells were maintained in growth medium. Transfection was carried out using the hsa‐miR‐29a‐3p miRVana mimic, the hsa‐miR‐29a‐3p miRVana inhibitor, and a non‐specific negative control (NC‐miRNA; Ambion), at a final oligonucleotide concentration of 20 nmol/mL. Transfections were performed using the RNAiMAX transfection reagent from Thermo Fisher. Opti‐MEM I (1X) Reduced Serum Medium (Gibco) was used to dilute both the RNAiMAX and the nucleic acids. The transfection protocol followed the manufacturer's instructions and was conducted in triplicate. After 48 hours of transfection, the cells were detached with trypsin, and cell pellets were stored at –80°C until RNA purification.

### 2.7 Flow cytometry analysis

Cytofluorimetric analysis was performed to assess the transfection efficiency in SK‐N‐BE cells. The BLOCK‐iT Alexa Fluor Red Fluorescent Oligo 20 µM, a non‐homologous oligonucleotide used as reliable indicator of lipid‐mediated transfection efficiency in RNAi experiments using the Lipofectamine RNAiMAX Transfection Reagent, was used (Invitrogen). This oligo does not exhibit homology to any known gene. Forty‐eight hours post‐transfection, the growth medium was removed, and the cells were washed twice with phosphate‐buffered saline. Fluorescence signals were analyzed using the phycoerythrin (PE) channel on a BD LSR Fortessa flow cytometer (BD Biosciences). Data were processed by BD FACSDiva software. In each analysis, 10^4^ events were acquired per sample.

### 2.8 DNA methylation profiling at CpG and non‐CpG moieties

CpG and non‐CpG DNA methylation was assessed by bisulphite DNA modification and Sanger sequencing using the HS (human) and MM (mouse) MIR29BisF1 and MIR29BisR1 non‐CpG methylation‐insensitive primers (MIPs) listed in Table 1. These primers allow the unbiased amplification of the *miR‐29a* genomic DNA sequences with unpredicted non‐CpG methylation. Briefly, DNA was extracted using the DNeasy Blood and Tissue Kit and the Qiacube and bisulphite‐treated by the EpiTect Bisulphite kit (Qiagen). Modified DNA was amplified with the PCRBIO Ultra Polymerase (PCRBIO) and the PCR products were cloned using the TA Cloning Kit (Thermo). At least 20 clones per experimental condition were analyzed, using M13 primers for the Sanger sequencing with the ABI PRISM 3130*xl* genetic analyzer (Thermo Fisher). For each experimental sample, methylation percentage of every single cytosine was calculated as the number of methylated cytosines divided by the number of sequenced clones x 100.^48^ Average methylation was calculated as the total of all‐C, CpG, and non‐CpG methylated moieties over the number of all‐C, CpG, and non‐CpG cytosine moieties. Overall methylation was calculated as the total of all‐C, CpG, and non‐CpG methylated moieties over the number of total (all‐C) cytosine moieties. The primers used allowed us to assess the methylation status of the minus (3′ → 5′) DNA strand, from which the miRNA sequence is transcribed. Positive and negative controls, aimed at checking conversion efficiency, were performed as described.^22^, ^48^

### 2.9 Statistical analysis

Statistical analyses were performed using one‐way or two‐way analysis of variance followed by a Tukey post hoc test to evaluate miRNA and mRNA expression. Contingency tables and Fisher exact tests were used for DNA methylation analysis. All histograms show the mean value ± standard error of the mean. Asterisks in figures indicate statistically significant differences. All the statistical analyses were computed using SPSS software.

## 3 RESULTS

### 3.1 miRNA profiling in SK‐N‐BE neuroblastoma cells

The SK‐N‐BE human neuroblastoma cell line has been used as a “first line” experimental model for the initial screening of miRNA expression. Cells were cultured for 48 hours in control and SAM‐supplemented medium and the miRNAs were retrotranscribed to assess their expression through a PCR miRNA array. Figure S1 in supporting information shows the miRNA expression profiles, and the molecular pathways and associated processes obtained after the KEGG (Figure S1a) and GO analyses (Figure S1b). The *miR‐29a* is highlighted by the blue boxes. The table in Figure S1c shows the eight miRNAs we selected for further confirmation by RT‐qPCR, based on a fold‐change threshold of < 0.5 or > 2. These include: four miRNAs upregulated in SAM‐supplemented cells versus Ctrl (miR‐7‐5p, miR‐29a‐3p, miR‐222‐3p, miR‐155‐5p); three miRNAs downregulated in SAM‐supplemented cells versus Ctrl (miR‐15a‐5p, let‐7g‐5p, miR‐17‐5p); one miRNA non‐modulated in SAM‐supplemented cells versus Ctrl (miR‐126‐3*p*).

After this screening and the PCR confirmation of up‐ or downregulation, we focused on *miR‐29a* because of its association with AD,^39^ and DNA methylation,^45^ as well as for its unexpected inverse modulation correlated with the DNA methylation induced by SAM supplementation.

### 3.2 miR29a modulation in AD models

**FIGURE 1:**
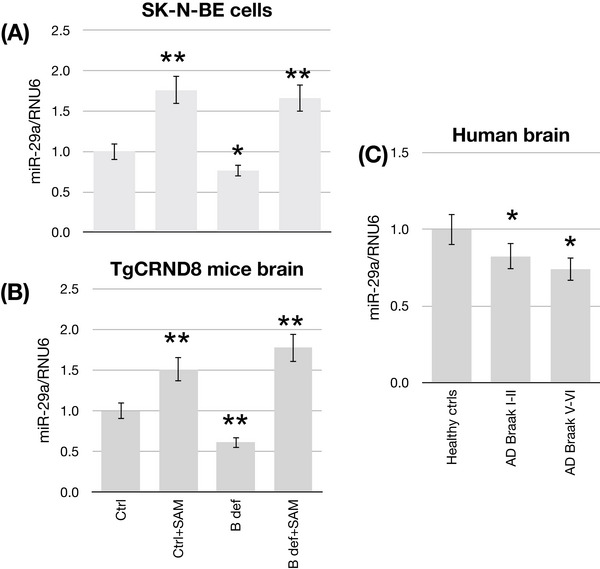
miR‐29a expression in AD models. Effects of hypermethylating (SAM‐supplementation) and hypomethylating (B‐deficient) treatments in SK‐N‐BE cell line (A) and TgCNRD8 mice (B). miR‐29a expression in *post mortem* human brain samples (C). Bar plots show the relative amounts of the miR‐29a cDNA normalized to the mean RNU6‐SNORD68 internal reference obtained using quantitative reverse transcription polymerase chain reaction on the *y* axis. Data represent the mean ± standard error of the mean. *p < 0.05; **p < 0.01. Cell cultures, *N* = 3; mice, *N* = 12; human samples, *N* = 15. AD, Alzheimer's disease; SAM, S‐adenosylmethionine.

The expression of *miR‐29a* was assessed by RT‐qPCR analysis in both SK‐N‐BE cells and in TgCRND8 mice under conditions of induced hypomethylation (B vitamin deficiency) and hypermethylation (SAM supplementation). The histograms in Figure 1 show that *miR‐29a* expression is induced by SAM supplementation and inhibited by B vitamin deficiency both in SK‐N‐BE cells (Figure 1A) and in mice brains (Figure 1B). *miR‐29a* upregulation in SAM‐supplemented cells and mice shows similar magnitude (≈ 50% increase) regardless of the concomitant B vitamin deficiency. Downregulation in B vitamin deficiency is ≈ 30% in cells and 40% in mice brains.

Additionally, *miR‐29a* expression was evaluated in *post mortem* brain tissue (prefrontal cortex) of healthy controls, AD patients at Braak stage I and II, and AD patients at Braak stage V and VI. The results (Figure 1C) confirmed that, as expected, *miR‐29a* is significantly downregulated in AD.

### 3.3 miR29a gain and loss of function analysis

Because we previously demonstrated^20^, ^23^ that *BACE1* mRNA was inversely regulated by B vitamin deficiency and SAM supplementation in respect to what we observed for *miR‐29a* (i.e., BACE1 is upregulated in B vitamin deficiency and downregulated by SAM), and considering it is targeted by this miRNA, we performed gain‐ and loss‐of‐function assays to mechanistically link these RNA expression results.

**FIGURE 2:**
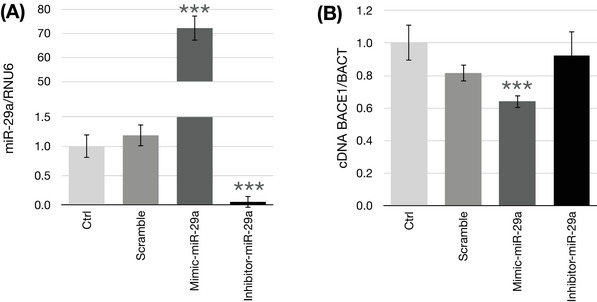
miR‐29a transfection in SK‐N‐BE cells. Expression of miR‐29a in SK‐N‐BE cells transfected for 48 hours with 20 µM mimic‐ or inhibitor‐miR29a (A). Effects of miR‐29a transfections on BACE1 mRNA expression (B). Bar plots show the relative amounts of the target genes normalized to the mean β‐ACTIN‐GAPDH internal reference obtained using quantitative reverse transcription polymerase chain reaction on the *y* axis. Data represent the mean ± standard error of the mean. ***p < 0.001; *N* = 3.

Figure 2A shows the effect of transfecting SK‐N‐BE cells with *miR‐29a* mimic or inhibitor sequences, resulting in significantly increased and decreased (respectively) intracellular *miR‐29a* levels.

Fluorescence‐activated cell sorting (FACS) analysis confirmed transfection efficiency in SK‐N‐BE neuroblastoma cells, showing that ≈ 70% of the treated cell population was successfully transfected (Figure S2 in supporting information).

The effect of the transfections on *BACE1* expression is shown in Figure 2B. Transfection with the miR‐29a mimic led to the expected reduction in *BACE1* mRNA levels. However, no significant *BACE1* upregulation was observed when the *miR‐29a* inhibitor was used. A similar expression pattern was observed for two other known targets of *miR‐29a*, *TET1* and *TNFR1* (Figure S3 in supporting information).

### 3.4 miR29a DNA methylation in AD models

**FIGURE 3:**
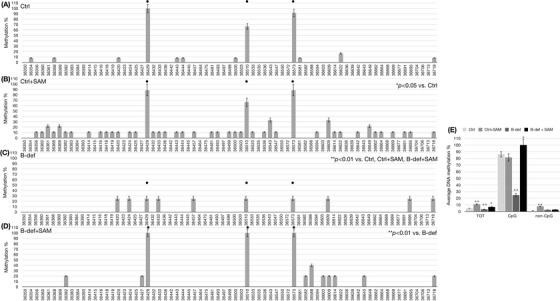
miR‐29a methylation in SK‐N‐BE cells. miR‐29a DNA CpG and non‐CpG methylation patterns in SK‐N‐BE cells. Histograms in (A) Ctrl, (B) Ctrl+SAM, (C) B vitamin deficiency, and (D) B‐def + SAM show methylation % (*y* axis) of each cytosine; labels on *x* axis indicate the cytosine position on the reference sequence of the DNA locus hosting the miR‐29a sequence. CpG cytosines are indicated by a dot over the related columns. Data represent the mean ± SEM *p < 0.05; ** *p* < 0.01; *N* = 3. Histogram in (E) shows the average methylation % over the total cytosines (*y* axis) as derived from the respective data and grouped in all the cytosines (TOT), CpG moieties (CpG), and non‐CpG moieties (non‐CpG). Data represent the mean ± SEM versus the related controls (light gray columns). *p < 0.05; ** *p* < 0.01; *N* = 3. SAM, S‐adenosylmethionine; SEM, standard error of the mean.

**FIGURE 4:**
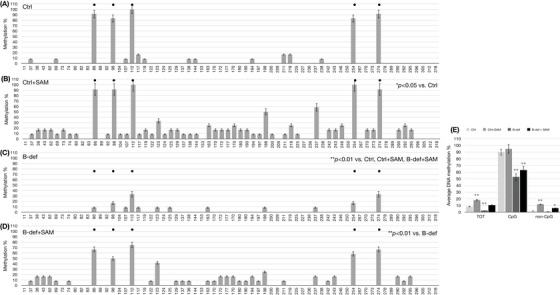
miR‐29a methylation in TgCRND8 mice. miR‐29a DNA CpG and non‐CpG methylation patterns in brain (prefrontal cortex) from TgCRND8 mice. Histograms in (A) Ctrl, (B) Ctrl+SAM, (C) B vitamin deficiency, and (D) B‐def + SAM show methylation % (*y* axis) of each cytosine; labels on *x* axis indicate the cytosine position on the reference sequence of the DNA locus hosting the miR‐29a sequence. CpG cytosines are indicated by a dot over the related columns. Data represent the mean ± SEM. *p < 0.05; ** *p* < 0.01; *N* = 3. Histogram in (E) shows the average methylation % over the total cytosines (*y* axis) as derived from the respective data and grouped in all the cytosines (TOT), CpG moieties (CpG), and non‐CpG moieties (non‐CpG). Data represent the mean ± SEM vs. the related controls (light gray columns) *p < 0.05; ** *p* < 0.01; *N* = 3. SAM, S‐adenosylmethionine; SEM, standard error of the mean.

**FIGURE 5:**
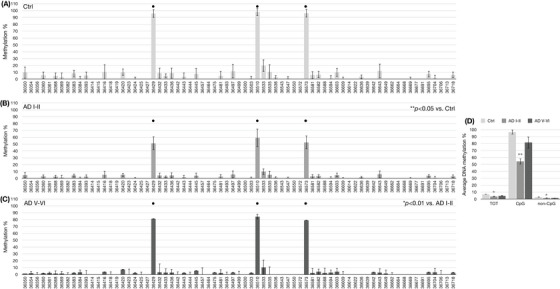
miR‐29a methylation in *post mortem* human brain. miR‐29a DNA CpG and non‐CpG methylation patterns in *post mortem* human brain (prefrontal cortex) from healthy and AD subjects. Histograms in (A) Healthy controls, (B) AD Braak stage I‐II, and (C) AD Braak stage V‐VI show methylation % (*y* axis) of each cytosine; labels on *x* axis indicate the cytosine position on the reference sequence of the DNA locus hosting the miR‐29a sequence. CpG cytosines are indicated by a dot over the related columns. Data represent the mean ± SEM. *p < 0.05; ** *p* < 0.01; *N* = 3. Histogram in (E) shows the average methylation % over the total cytosines (*y* axis) as derived from the respective data and grouped in all the cytosines (TOT), CpG moieties (CpG), and non‐CpG moieties (non‐CpG). Data represent the mean ± SEM versus the related controls (light gray columns). *p < 0.05; ** *p* < 0.01; *N* = 3. AD, Alzheimer's disease; SEM, standard error of the mean.

Due to the characteristic regulation of *miR‐29a* expression observed under B vitamin deficiency and SAM supplementation, we assessed the methylation profile of the *miR‐29a* locus by bisulphite assay, followed by cloning of the PCR products and Sanger sequencing. To assess both CpG and non‐CpG methylation, we used PCR primers unbiased versus non‐CpG methylation (MIPs).^48^ The methylation pattern of *miR‐29a* was assessed in DNA isolated from SK‐N‐BE cells (Figure 3), prefrontal cortex of TgCRND8 mice (Figure 4), and *post mortem* prefrontal cortex from human brain samples (Figure 5).

Histograms on the left side of Figures 3 through 5 report the methylation percent for each CpG and non‐CpG (CpA, CpT, CpC) cytosines in the DNA locus coding for the *miR‐29a*. For the human sequence, this corresponds to cytosines 36350 through 36718 and for the mouse sequence, cytosines 11 through 318. The CpG sites present in the region are marked by a dot over the corresponding columns. The histograms on the right side of Figures 3 through 5 display the average methylation levels of three groups of cytosines: all the cytosines (TOT), CpG cytosines, and non‐CpG cytosines.

SAM supplementation induces significant hypermethylation (*p *< 0.05), particularly at the non‐CpG moieties in both SK‐N‐BE cells (Figure 3B) and mouse cortex (Figure 4B) compared to the control conditions (Figures 3A and 4A, respectively). This effect is also evident in Figures 3E and 4E, which show no changes in overall CpG methylation but increased overall non‐CpG methylation (middle gray columns). When treated with B vitamin deficient cell culture medium or rodent diet, both cells (Figure 3C) and mouse brains (Figure 4C) exhibit reduced CpG methylation (*p *< 0.01) compared to controls (Figures 3A and 4A), whereas non‐CpG methylation remains at levels comparable to the control (Figures 3A and 4A). In contrast, when SK‐N‐BE cells and TgCRND8 mice are treated with the combination of B vitamin deficiency and SAM supplementation, CpG and non‐CpG methylation in cells return to control‐like levels (Figure 3D and 3E). However, in mouse brains, only a partial recovery in CpG methylation is observed (Figure 4D and 4E, *p *< 0.05), while non‐CpG methylation increases significantly beyond control levels (Figure 4D and 4E, *p *< 0.01).

Overall, DNA methylation is positively correlated with *miR‐29a* expression, with higher methylation levels observed under conditions that promote miR‐29a expression.

Human brain samples, derived from subjects with unknown MP and methylation status until now, show hypomethylation at CpG moieties at Braak stages I and II compared to controls (Figure 5B and 5E, *p *< 0.05). Braak stage V and VI CpG methylation shows an intermediate level (Figure 5C and 5E, *p *< 0.01). Non‐CpG methylation in these samples remains low and comparable to controls.

## 4 DISCUSSION

Recent evidence indicates that DNA methylation may play a central role in regulating miRNA expression.^25^, ^49^, ^50^, ^51^ The “one‐carbon metabolism” is the metabolic pathway responsible for regulating the DNA methylation pattern. This pathway involves several key components, including SAM—the main endogenous methyl donor—and several enzymatic cofactors belonging to the B vitamin group (B12, B6, folate).^52^

Alterations in one‐carbon metabolism along with hyperhomocysteinemia have been previously implicated in the development of dementia as well as AD.^18^, ^20^, ^53^ By studying the effect of one‐carbon metabolism alterations on miRNA expression, we identified *miR‐29a* as a promising candidate with a protective role in neurodegeneration. *miR‐29a* directly targets *BACE1* ^39^ which we have previously shown to be modulated by one‐carbon metabolism but in a methylation‐independent manner.^20^, ^23^

Transfection assays with miRNA‐specific mimics and inhibitors in a human neuroblastoma SK‐N‐BE cell line allowed us to verify the effects of *miR‐29a* modulation on its direct targets. Additionally, we assessed through the sodium bisulphite assay and DNA sequencing the *miR‐29a* methylation pattern under one‐carbon metabolism alterations. We found that increased CpG and non‐CpG methylation within the *miR‐29a* coding sequence is associated with the overexpression of the miRNA and with *BACE1* repression, and vice versa. Beyond their intracellular role in regulating of transcription and/or translation, miRNAs can be secreted outside the cells and are therefore retrieved in the extracellular fluids, including in the blood.^54^ This characteristic highlights *miR‐29a*’s potential as a new biomarker for AD, supported by the recent development of a rapid biosensor capable of detecting and quantifying the *miR‐29a* in body fluids.^55^ Notably, different miRNAs targeting genes involved in AD have been identified and studied for their molecular roles, aiming to disclose possible miRNA signatures associated with AD risk or diagnosis.^56^, ^57^ The identification of a panel of miRNAs as biomarkers could be therefore readily translated into clinical applications through the development of specific and, possibly, multiplex biosensors.

While the use of miRNAs as biomarkers is an evident and easily translatable application, the potential to target specific miRNAs for preventing or combating AD pathology is equally compelling. These therapeutic approaches could involve using miRNA inhibitors (miRNAi) to suppress deleterious miRNAs or administering “beneficial” miRNAs directly. Nanoparticle‐based delivery systems for miRNAs have already been proposed for the treatment of different diseases,^58^ including respiratory diseases,^59^ cardiovascular diseases,^60^ and mainly cancer.^61^ However, recent studies are also exploring the potential of delivering miRNAs to treat brain pathologies.^62^ Among the most promising advances are the non‐viral lipid nanoparticles,^63^ which have shown efficacy in crossing the blood–brain barrier.^64^ It has been already demonstrated that targeting a specific miRNA (miR‐17) in the brain of AD transgenic mice using lipid nanoparticles improves both the pathological phenotype and behavior.^65^ Identifying panels of miRNAs associated with AD could thus foster the development of such delivery systems for therapeutic applications. In this perspective, our findings offer a further new perspective on therapeutic miRNA modulation. The finding that *miR‐29a* is modulated by the one‐carbon metabolism, and the possibility that other miRNAs^25^ might undergo similar regulation, fosters the epigenetic intervention, through modulators of the one‐carbon metabolism, to drive the expression of methylation‐regulated miRNAs. In this context, the one‐carbon metabolism modulation, and in particular SAM supplementation, seems to acquire a central “pleiotropic” role in AD. In fact, we have previously demonstrated in AD experimental models that SAM supplementation can counteract key pathological molecular processes associated with neurodegeneration, including amyloid accumulation,^20^, ^23^ tau hyperphosphorylation,^66^ and oxidative stress.^67^ Furthermore, the potential for SAM to modulate neuroinflammation through the expression of cytokines regulated by DNA methylation^68^ is currently under investigation in our laboratory.

It is noteworthy that the DNA methylation–dependent regulation of *miR‐29a* expression appears to be counterintuitive compared to the classic inverse relationship in which high methylation corresponds to low mRNA expression. However, it is increasingly recognized—though often underrated—that methylation in regulatory regions and gene bodies can have opposing effects on expression regulation.^69^ Specifically, promoters methylation negatively affects mRNA expression by reducing the accessibility to the transcription factors (TFs), while gene body methylation may enhance mRNA expression. This enhancement occurs by reducing TF binding to alternative start sites and by increasing the elongation efficiency.^70^ A recent extensive analysis at genomic level confirms this evidence and highlights that gene expression is positively correlated to gene‐body methylation.^71^ Interestingly, another recent study provides evidence of a role for non‐CpG exons methylation,^72^ as also evidenced by our results. The functional role of gene‐body methylation has been proven in the regulation of mRNAs, as very recently demonstrated for the MGMT mRNA that was found positively correlated with body methylation and negatively correlated with promoter methylation in gliomas.^73^ However, this effect may be particularly relevant for ncRNA sequences, usually located in inter‐ and intragenic regions.^74^ A recent study, in fact, demonstrated the existence of a positive correlation between methylation at gene‐body level and circular‐RNA (circRNA) expression.^75^ These results suggest that epigenetic features may play an important role in the definition of the cell circRNA pool and very well match with our results on miR‐29a. Consequently, DNA methylation in gene bodies may acquire greater attention for its involvement in ncRNA expression, especially given advances in genomic approaches to the study of DNA methylation, allowing detection of methylation at regulatory and intragenic regions. Of course, we cannot conclude that this mechanism applies to all the genes as it probably depends on the specific sequence and on the presence and position of TF‐binding sites and alternative transcription start sites (TSS).

Finally, the methylation data reported here further highlight the presence of detectable non‐CpG methylation, which is also modulated by both hyper‐ and hypomethylating experimental conditions. Consistent with previous studies, it seems that SAM supplementation is capable of inducing DNA hypermethylation, particularly at non‐CpG sites.^14^, ^48^

In conclusion, the data reported here indicate that one‐carbon metabolism modulation contributes to amyloid processing by regulating the expression of the *miR‐29a*, which targets *BACE1*. Specifically, SAM supplementation induces *miR‐29a* expression through hypermethylation of its DNA sequence, thereby leading to a reduction in *BACE1* expression. Together with previous data showing that SAM supplementation can also reduce *PSEN1* expression via direct DNA methylation of its promoter,^17^, ^20^ the present findings contribute to building the hypothesis of a pleiotropic effect of SAM—and one‐carbon metabolism in general—in regulating molecular processes associated to neurodegeneration, as depicted in the graphical abstract.

These results strongly support the idea that *miR‐29a* and other miRNAs hold potential as biomarkers for AD. Moreover, it may be possible to modulate these miRNAs to treat or even prevent the pathology.

## AUTHOR CONTRIBUTIONS

Andrea Fuso, Marco Lucarelli, Tiziana Raia, and Luiza Diniz Ferreira Borges performed the experiments, and the mRNA expression and the DNA methylation analysis. Rosaria A. Cavallaro, Stefano Cinti, and Mariano Bizzarri contributed to the data analysis and the manuscript writing. Isidre Ferrer provided human brain samples. Marco Lucarelli and Andrea Fuso conceived and supervised the overall experimental design and prepared the manuscript for submission. All authors read and approved the final manuscript. This study was funded by Sapienza University, Projects #RM11916B88D5E704 and #AR123188B457AF5E, and by “Gnosis by Lesaffre.”

## CONFLICT OF INTEREST STATEMENT

Tiziana Raia, Luiza Diniz Ferreira Borges, Rosaria A. Cavallaro, Isidre Ferrer, Stefano Cinti, Mariano Bizzarri, and Marco Lucarelli have nothing to disclose. Andrea Fuso is a consultant at “Gnosis by Lesaffre.” Author disclosures are available in the supporting information

## ETHICS STATEMENT

Wild‐type and transgenic animals used in this study were bred at the Sapienza University of Rome. All procedures were carried out in accordance with the European Communities Council Directive (86/609/EEC 2010/63/EU) and were approved by the Italian Ministry of Health and by the local ethical committee. Work on human subjects: *Post mortem* adult brain samples used in this study were obtained from the Institute of Neuropathology and Brain Bank (HUB‐ICO‐IDIBELL Biobank) following the guidelines of the Declaration of Helsinki, and according to the Spanish and Catalonian Autonomous regulations on this matter, and the approval of the local Ethics Committee of the Bellvitge University Hospital.

## Supporting information



Supporting Information

Supporting Information

Supporting Information

Supporting Information

## Data Availability

The datasets used and/or analyzed during the current study are available from the corresponding author upon reasonable request.
