# Supplementary material for: One‐carbon metabolism modulates miR‐29a–DNA methylation crosstalk in Alzheimer's disease
Source: Alzheimers Dement. 2025 Sep 23;21(9):e70703. doi: 10.1002/alz.70703 (PMC12457075; doi:10.1002/alz.70703)
Supplement: Supplementary file 2 — Supporting Information [file ALZ-21-e70703-s002.pdf]

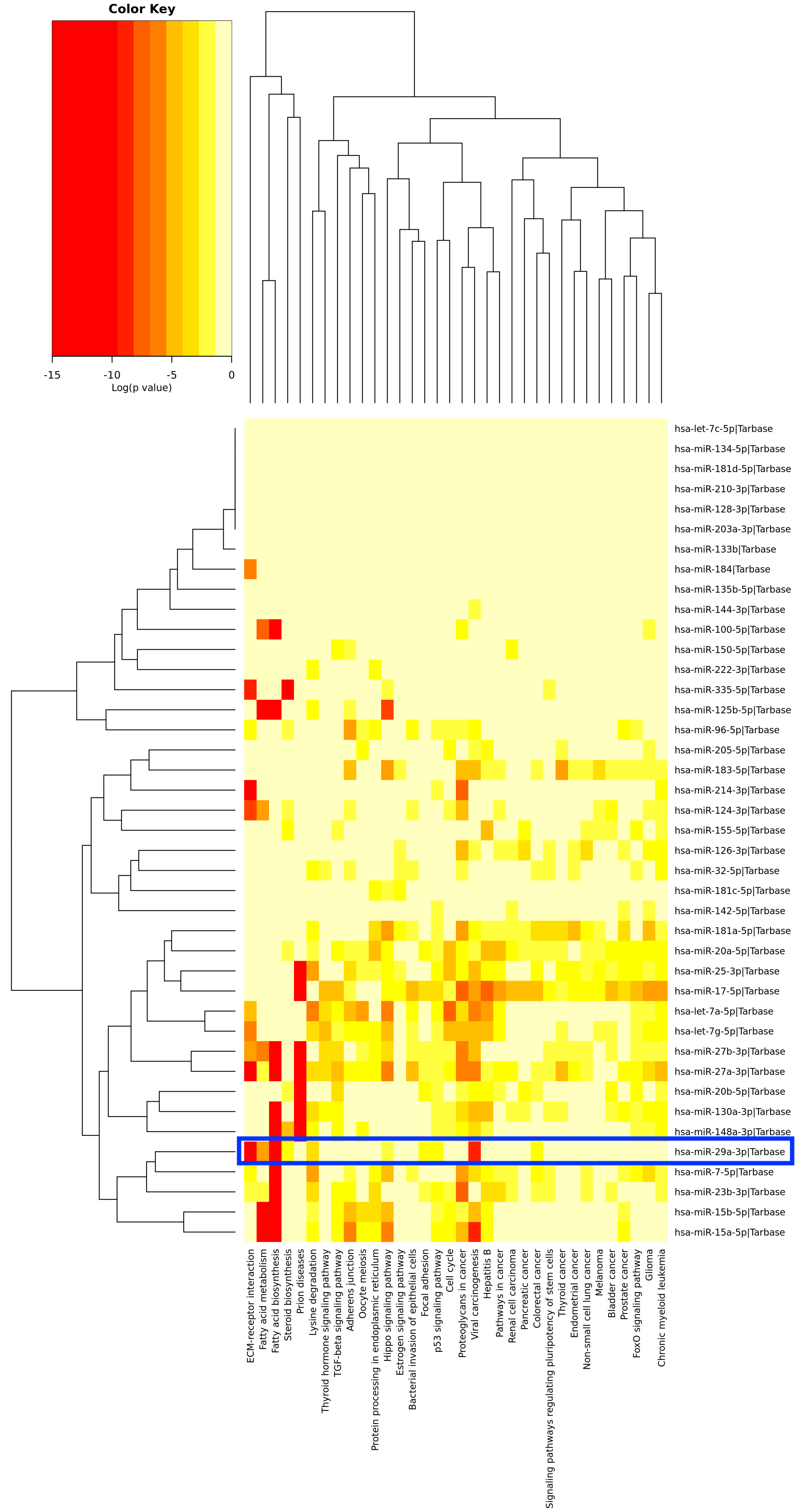

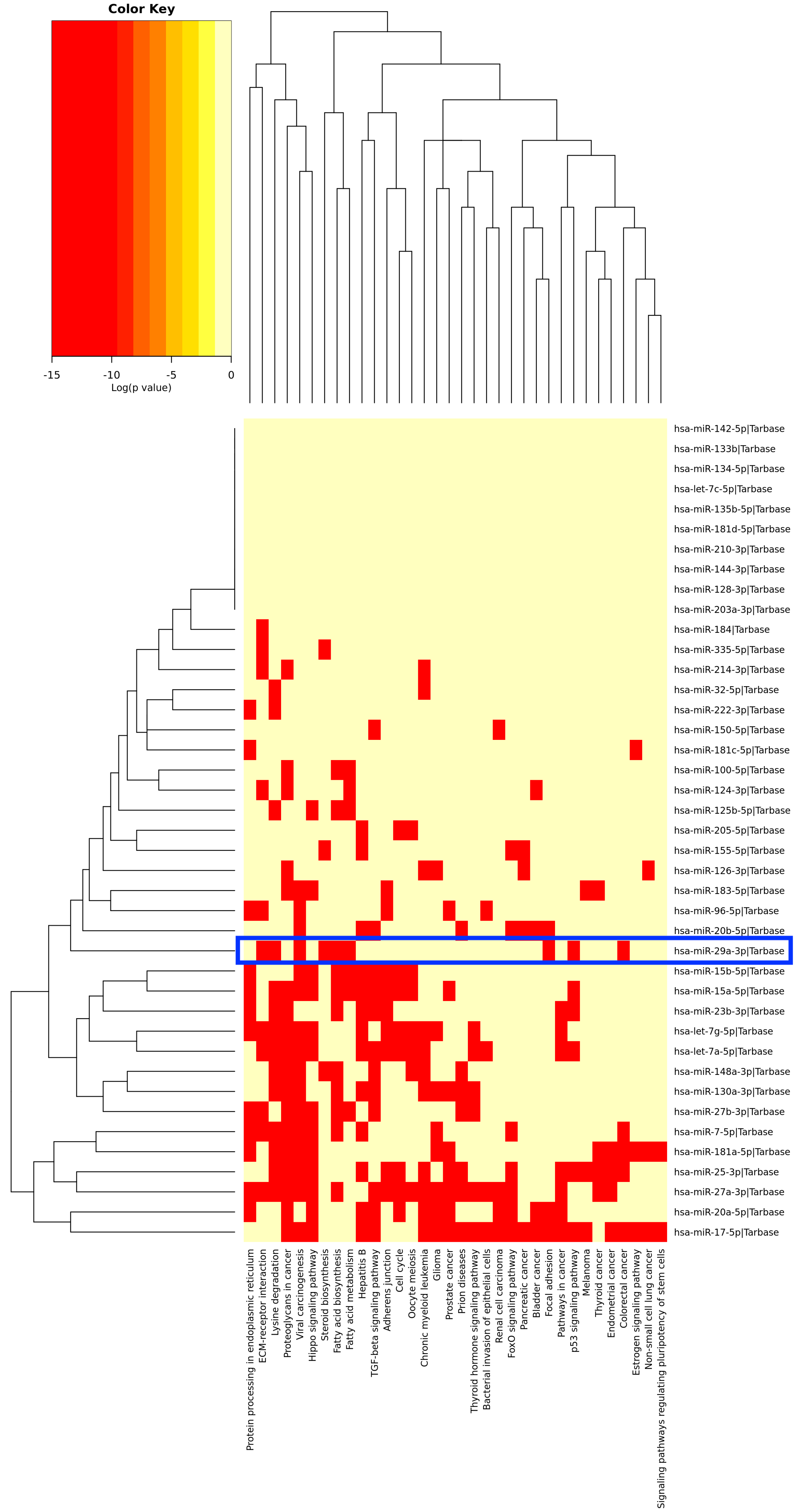

c

microRNA	Fold Change
hsa-miR-126-3p	/
hsa-miR-15a-5p	0,35
hsa-let-7g-5p	0,44
hsa-miR-7-5p	2,07
hsa-miR-29a-3p	3,99
hsa-miR-222-3p	3,98
hsa-miR-155-5p	3,85
hsa-miR-17-5p	0,14

Supplementary Figure 1: microRNA PCR array analysis.
The cluster-heat-map shows microRNAs grouped based on their involvement in the same cluster and/or for having a role in the same biological process with a high (red) or low (yellow) p-value obtained from "KEGG" (a) and "GO" (b) analyses of the molecular pathways and associated processes. Suppl. Fig. 1c highlights the subgroup of eight human microRNA selected after SAM treatment of SK-N-BE cells compared to the control by microRNA PCR array. In these experiments miR-126-3p was not expressed in these analysis's conditions. miR-15a-5p, miR-let-7g-5p and miR-17-5p were down-regulated with fold changes below 0.5. miR-7-5p, miR-29a-3p and miR-155-5p were up-regulated, showing a fold changes greater than 2.
