# Supplementary material for: One‐carbon metabolism modulates miR‐29a–DNA methylation crosstalk in Alzheimer's disease
Source: Alzheimers Dement. 2025 Sep 23;21(9):e70703. doi: 10.1002/alz.70703 (PMC12457075; doi:10.1002/alz.70703)
Supplement: Supplementary file 3 — Supporting Information [file ALZ-21-e70703-s004.pdf]

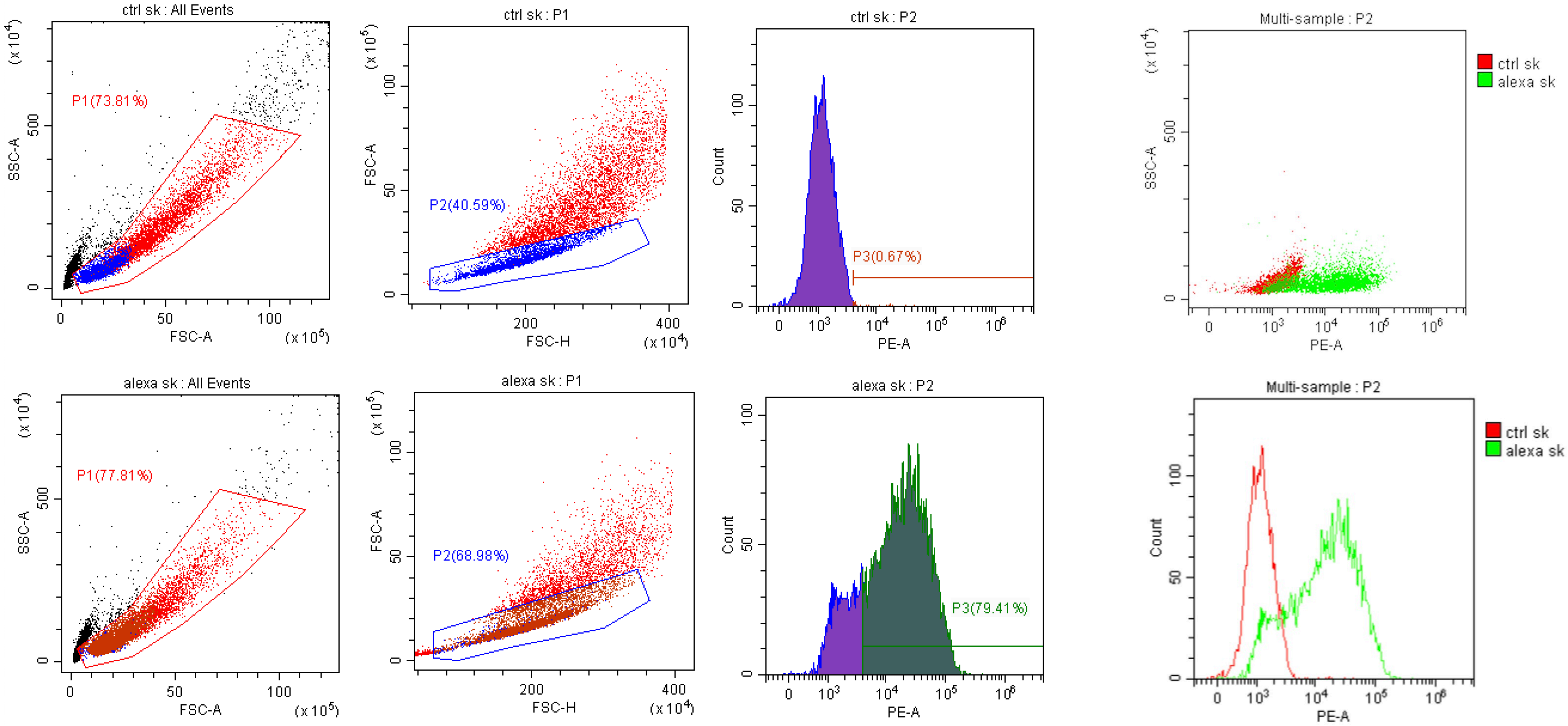

Supplementary Figure 2: Analysis of the neuroblastoma cell line population in transfection assays.
FACS gating strategy for Block-iTTM Alexa FluorTM Red Fluorescent Control transfected cells (alexa sk) compared to untreated cells (ctrl sk). The P2 population represents the frequency of single cells. The green peaks in the right histograms represent the positive transfected cells compared to the non-transfected ones represented by the red peak.
