# Supplementary material for: One‐carbon metabolism modulates miR‐29a–DNA methylation crosstalk in Alzheimer's disease
Source: Alzheimers Dement. 2025 Sep 23;21(9):e70703. doi: 10.1002/alz.70703 (PMC12457075; doi:10.1002/alz.70703)
Supplement: Supplementary file 4 — Supporting Information [file ALZ-21-e70703-s003.pdf]

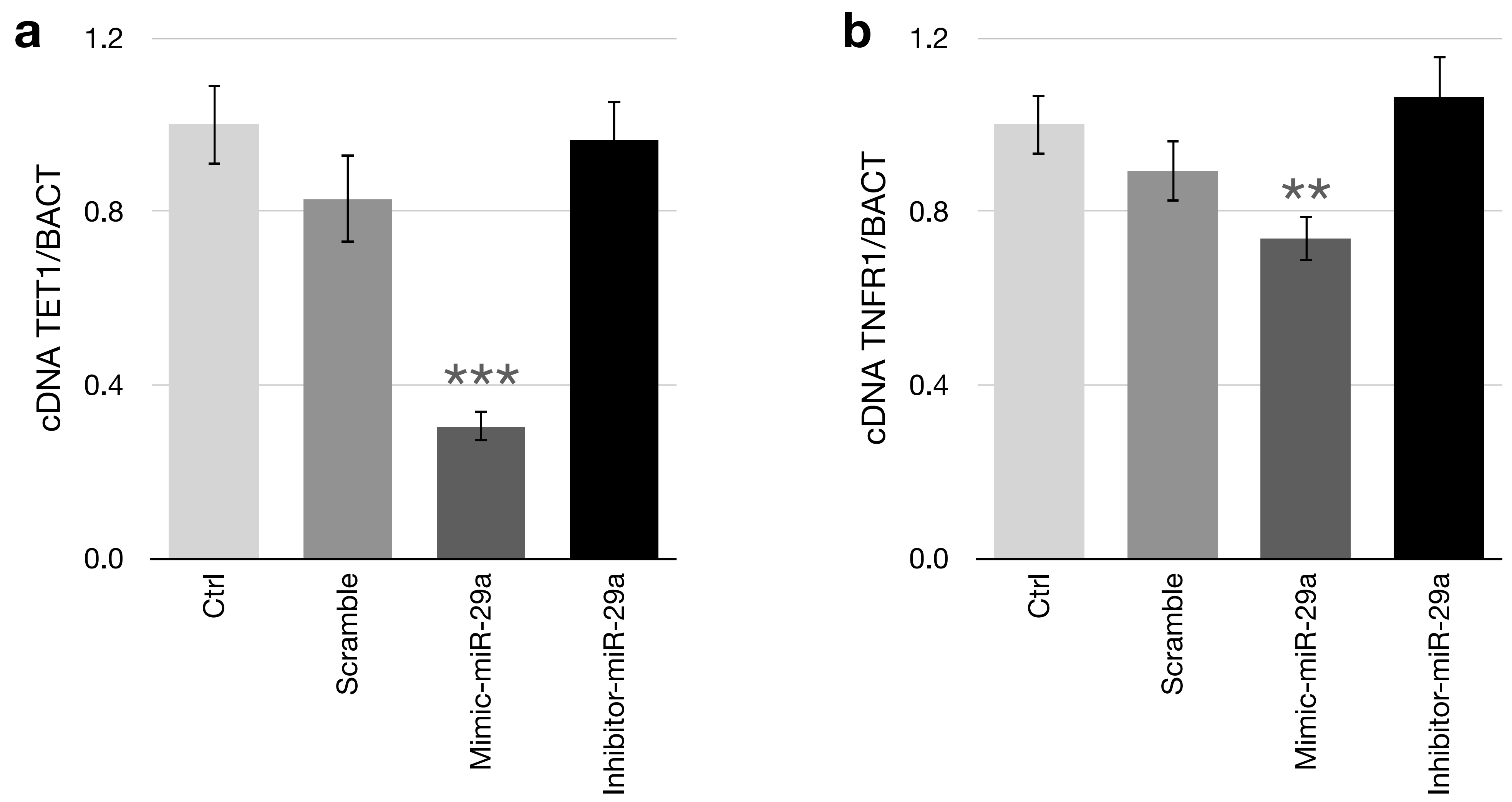

Supplementary Figure 3: Effects of mimic- and inhibitor- miR29a transfection in SK-N-BE cell line. Expression of miR-29a target genes (mRNA) in SK-N-BE cells transfected for 48 h with 20μM mimic- and inhibitor- miR29a. mRNA expression of TET1 (a) and TNFR1 (b). Bar plots show the relative amounts of the target genes normalized to the mean β-ACTIN internal reference obtained using qRT-PCR on the y-axis. Data represent the mean ± SEM **p<0.01; ***p<0.001; N = 3.
